# Cumulative *Plasmodium falciparum* infections do not drive long-term telomere shortening in Kenyan children

**DOI:** 10.3389/fcimb.2026.1750881

**Published:** 2026-05-13

**Authors:** Aurelie Miglar, David Amadi, David Grannas, Linnea Widman, Jedida Mwacharo, Jennifer Musyoki, Oscar Kai, Philip Bejon, Alex Maccharia, Thomas N. Williams, Anna Färnert, Muhammad Asghar, Francis M. Ndungu

**Affiliations:** 1 Division of Infectious Diseases, Department of Medicine Solna, Karolinska Institutet, Stockholm, Sweden; 2 Department of Infectious Diseases , Karolinska University Hospital, Stockholm, Sweden; 3 Center for Molecular Medicine , Karolinska Institutet, Stockholm, Sweden; 4 Kenya Medical Research Institute, Centre for Geographical Medicine Research Coast, Kilifi, Kenya; 5 Department of Biostatistics, Environmental Medicine, Karolinska Institutet, Stockholm, Sweden; 6 Nuffield Department of Medicine, Oxford University, Oxford, United Kingdom; 7 Institute of Global Health Innovation, Department of Surgery and Cancer, Imperial College, London, United Kingdom; 8 Centre for Tropical Medicine and Global Health, University of Oxford, Oxford, United Kingdom; 9 Department of Biomedicine, National University of Sciences and Technology, Islamabad, Pakistan

**Keywords:** cellular ageing, children, malaria, *Plasmodium falciparum*, telomere length (TL), Sub-Saharan Africa, asymptomatic *P. falciparum* infection, asymptomatic malaria

## Abstract

**Background:**

Malaria is a severe and fatal disease in the non-immune, but severity lessens with increasing exposure. Children in endemic areas are at a particularly high-risk for malaria, often experiencing multiple sequential clinical and asymptomatic *P. falciparum* infections annually. Whether the associated persistent cellular activation has additional hidden costs, as reflected by cellular aging, has not been studied.

**Methods:**

We measured correlations between telomere length (TL) in cross-sectional blood samples (2007, 2010 and 2013) and cumulative malaria and *P. falciparum* infection episodes in children from two longitudinal cohorts under active weekly surveillance for febrile malaria. TL was quantified by qPCR in 218 children from Junju, an area of moderate transmission, and 90 age-matched controls from Ngerenya, where *P. falciparum* transmission is no longer endemic.

**Results:**

Although TL declined with age (independently of cumulative malaria exposure; linear effects model, p>0.05) in both cohorts, the decline was more pronounced in those with longer TL at baseline. Asymptomatic *P. falciparum* parasitaemia, at the time of the cross-sectional survey, was associated with a positive change in TL of 0.22 kb (95% confidence interval 0.02-0.42, p=0.03).

**Conclusion:**

Overall, repeated asymptomatic and symptomatic *P. falciparum* malaria episodes were not associated with TL shortening in the children in our cohorts. The apparent increase in length with asymptomatic malaria was of marginal statistical significance and could have been a chance finding. Alternatively, the suppression of cellular activation and proliferation, recently reported in asymptomatic *Plasmodium* spp infections, preserves TLs from degrading.

## Introduction

Despite recent progress in malaria control, including the introduction of routine immunisation with RTS,S, malaria resurged globally, with case numbers increasing from 249 million in 2022 to 263 million in 2023 (WHO, 2024). In malaria-endemic countries, both symptomatic episodes and asymptomatic infections are common in children (Botwe et al., 2017). Repeated exposure leads to partial immunity that reduces the severity of clinical disease, however, individuals frequently experience persistent, often chronic, asymptomatic infections throughout life, suggesting that naturally acquired immunity is not sterlile (Langhorne et al., 2008). In areas of high to moderate transmission, the burden of severe, life-threatening malaria and associated mortality is most pronounced in infants and young children (Brooker et al., 2017). Severe malaria can affect multiple organ systems, including the central nervous system (cerebral malaria), the pulmonary system (respiratory distress), the renal system (acute renal failure), the hematopoietic system (severe anaemia), and is frequently associated with metabolic acidosis (Bartoloni and Zammarchi, 2012). Beyond acute morbidity, malaria exposure has been linked to impaired neuro-cognitive and socio-economic development, and poor school performance in children, with potentially other long-term hidden health-costs for affected individuals (Vitor-Silva et al., 2009; Fink et al., 2013).

Evidence from animal and clinical studies suggests that malaria may have lasting biological effects beyond acute infection. In birds, chronic asymptomatic malaria reduces life span and fitness by accelerating telomere shortening (Asghar et al., 2015). In humans, a single acute malaria episode in Swedish adult travellers returning from the tropics was associated with accelerated telomere shortening in peripheral blood leukocytes, which reversed within a few months following treatment (Asghar et al., 2018). A more modest but similarly reversible reduction in telomere length (TL) was also observed in malaria-naïve volunteers undergoing controlled human malaria infection (CHMI) (Miglar et al., 2021). However, the extent to which repeated clinical episodes and persistent asymptomatic infections influence telomere dynamics in children living in endemic areas remains poorly understood (Wakai et al., 2025).

Telomeres are non-coding repetitive DNA sequences that protect chromosome ends from inappropriate DNA damage recognition and progressive sequence loss (Jacobs, 2013). In telomere biology, it is important to distinguish between telomere length (TL) as the length of telomeric DNA measured at a given time point, the telomere shortening rate as dynamic indicator of longitudinal attrition, and telomerase activity, which reflects the ability of telomerase to mitigate telomere loss by adding telomeric repeats, particularly in stem and activated immune cell populations (Hoare et al., 2010; Lopez-Otin et al., 2013). Telomere shortening is a hallmark of ageing and is closely linked to cellular senescence (Shawi and Autexier, 2008; Hoare et al., 2010; Lopez-Otin et al., 2013). In both humans and animals, TL and the rate of telomere attrition have been associated with host fitness, disease risk, including cancer, and life expectancy (Cawthon et al., 2003; Wu et al., 2003; Yang et al., 2009). Together, these studies highlight the importance of understanding how lifelong exposure to infections such as malaria influences telomere dynamics and long-term health.

To separate the impact of malaria from other environmental variables would require data from individuals with similar characteristics, such as environment, genetics and socio-economic level, but with differential exposure to malaria. Such data is scarce in natural populations. The present study addresses this gap by examining the association between cumulative malaria exposure and telomere shortening over time, using blood samples and detailed weekly records of children under longitudinal surveillance for malaria for over 10 years. The study was conducted in two coastal regions of Kenya inhabited by the Miji Kenda people, with similar cultural practices, nutrition, and socio-economic characteristics but different malaria exposures (Ndungu et al., 2012; Illingworth et al., 2013). As TL was measured in peripheral blood leukocytes at three cross−sectional time points, our analyses primarily address longer−term patterns and associations rather than the previously reported short−term TL dynamics following acute episodes.

## Methods

### Ethical approvals

The study was approved by the KEMRI Scientific and Ethics Review Unit (Prot. no. KEMRI/SERU/CGMR-C/017/3149), and by the Swedish Ethical Review Authority (Dr. nr. 2008/998-31–3 and 2019-05746). Informed written consent was given by the parents of all the participants, or their guardians.

### Study population and procedure

The study cohorts in Junju and Ngerenya are in Kilifi County, on the Indian Ocean coast of Kenya and are nested within the Kilifi Health and Demographic Surveillance System (HDSS) that includes 350,000 participants (Cowgill et al., 2006; Olotu et al., 2010). Despite their geographic proximity, which measures about 40 km, similarities in ethnic make-up, social and economic structures, nutrition, and culture, the populations living in Junju and Ngerenya are exposed to different levels of malaria (Mwangi et al., 2005; Bejon et al., 2007; Ndungu et al., 2012; Illingworth et al., 2013). Malaria remains endemic in Junju with moderate transmission during the biannual malaria seasons (May-July and October-December), and with an average of 27% prevalence of asexual *P. falciparum* parasitaemia during the dry season in the years 1998 -2016 (Mwangi et al., 2005; Ndungu et al., 2012; Illingworth et al., 2013; Muthui et al., 2019). In contrast, in Ngerenya, malaria transmission fell to low levels in the early 2000s, with few reported cases of clinical or asymptomatic *Plasmodium* infections, as determined through both passive surveillance and annual cross-sectional qPCR-based surveys (Ndungu et al., 2012). Children from both cohorts are routinely recruited at birth into long-term longitudinal studies for malaria immunology. Ngerenya was under active weekly surveillance for malaria between 1998 and 2007, before shifting to passive surveillance in 2014 owing to the scarcity of malaria infections. Junju remains under active weekly surveillance for malaria since 2005. During the weekly visits, field workers measure axillary body temperature, *P. falciparum* positivity by rapid diagnostic test (RDT), and document the history of fever. In the event of fever, or its recent history and a positive RDT, a venous blood sample is drawn, and the child is treated for malaria.

Annual cross-sectional surveys are conducted in both cohorts in March, following a three-to-four-month dry period with minimal *P. falciparum* transmission, where anthropometric and parasitological data are collected, alongside with a 5 ml venous blood sample, which was collected if the child had fever (≥37.5˚C). The blood is processed into peripheral blood mononuclear cells (PBMC) and plasma and stored in liquid nitrogen and -80˚C, respectively, until use. An additional whole blood pellet (0.3ML) in EDTA is stored for DNA extraction.

**Figure 1:**
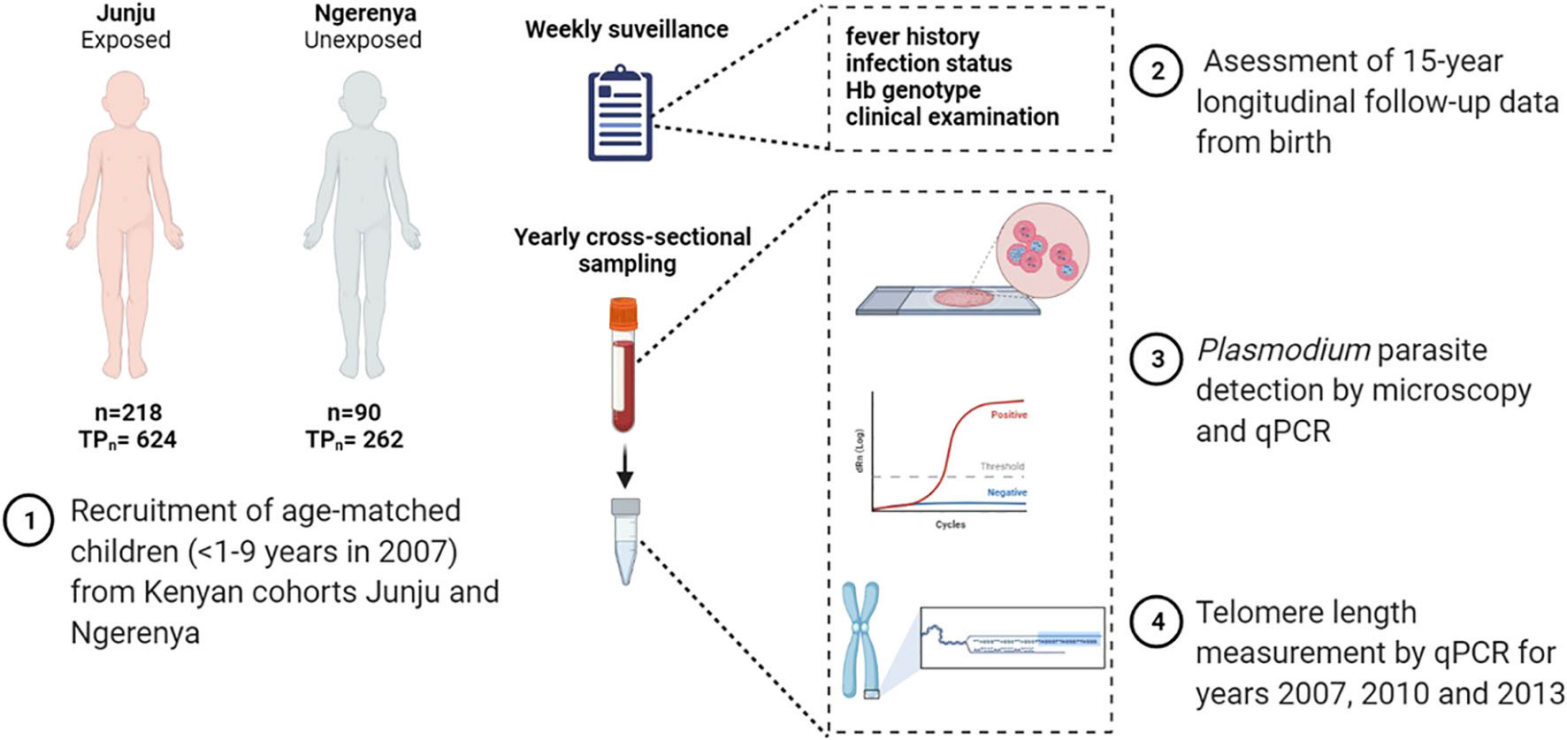
Study workflow.

The data and samples analyzed in this study are a subset of both, the annual surveys and longitudinal malaria surveillance conducted between 2005 and 2018 for Junju, and between 2002 and 2013 for Ngerenya. Inclusion criteria for this study were the age of ≤1–9 years in year 2007 (first study time-point) from Junju and age-matched children from Ngerenya. Telomere length was analyzed on three cross-sectional time points (years 2007, 2010 and 2013). The study design and workflow are presented in **Figure 1**.

### Case definition of malaria

Symptomatic malaria was defined as fever (temperature ≥ 37.5 °C) and parasite detection by microscopy or qPCR. A new episode of symptomatic malaria was defined as subsequent febrile parasitemic episode that occurred later than 7 days after the previous acute malaria illness. Asymptomatic infections were defined as axillary temperature <37.5 °C and *Plasmodium* sp. parasitaemia detected by RDT, microscopy and/or qPCR. Febrile children with axillary temperature ≥ 37.5 °C and who were parasite negative by RDT, microscopy and/or qPCR are referred to as non-malarial fever cases, while parasite negative individuals without fever are referred to as healthy.

### Parasite detection by microscopy and qPCR

Parasite detection in active surveillance data (weekly surveys) was based on RDT and microscopy. For the Junju cohort, parasitaemia was estimated based on actual leukocyte count measured for each blood smear (Olotu et al., 2010). Asexual parasite density was counted against 200 WBCs, and symptomatic parasite density (parasite/μl of whole blood) was calculated as follows: (number of parasites counted/WBC counted) × WBC count/µl of participant. Parasitic assessment in passive surveillance data (yearly surveys) was based on performing a *Plasmodium* species-specific real-time PCR assay targeting the multi-copy 18S rRNA gene using an ABI TaqMan 7500 instrument (Applied Biosystems) (Shokoples et al., 2009). In our analysis, we included only *P. falciparum* species parasitaemia, due to very low prevalence of other *Plasmodium* species.

### Genotyping for sickle cell trait and α-thalassaemia

DNA was extracted from whole blood using Qiagen™ DNA blood mini-kits (Qiagen Crawley, United Kingdom) and genotyped for sickle cell trait (AS) and disease (SS) and α-thalassaemia using PCR, as described previously (Chong et al., 2000; Waterfall and Cobb, 2001).

### DNA extraction and TL measurement

DNA was extracted from blood pellets (30uL) using an automated Qiaxtractor (Qiagen) and stored at -20 °C. Quantification was done using NanoDrop™ 2000 Spectrophotometer.

TL was assessed by quantitative real-time PCR (qPCR) following the method described in Asghar et al (Asghar et al., 2018). DNA was diluted to approx. 2 ng/μl to measure TL on an ABI 7500 real-time PCR system (Applied Biosystems) (Cawthon, 2002). A telomere-specific amplicon set of primers (TEL1, TEL2) was used to quantify TL together with a single-copy gene amplicon set of primers (HBG1, HBG2), to control for total amount of DNA in each reaction (Cawthon, 2002). The PCR reaction was prepared in a 25 µl final volume to the following concentrations: 1 × Platinum^®^ Quantitative PCR SuperMix-UDG (Invitrogen, cat # 1730025), 0.1 pmol of each primer; 0.1x ROX reference dye (Invitrogen, cat # 1730025) and 5 µl template DNA. Primer sequences for TEL1, TEL2 are found in Cawthon et al (Cawthon, 2009), and sequences for single-copy gene primers are described elsewhere (Cawthon, 2002; Zanet et al., 2013). Thermal profile of telomere PCR included an incubation period for 2 min at 50 °C, activation of the polymerase for 10 min at 95 °C, followed by 30 cycles of 95 °C for15 s, 53 °C for 45 s and 72 °C for 45s. The thermal profile for single copy PCR included an incubation period for 2 min at 50 °C, activation of the polymerase for 10 min at 95 °C, followed by 40 cycles of 95 °C for 15 s, 58 °C for 45 s and 72 °C for 45 s. A melt curve step was included in each PCR setup including: 95 °C for 15 s, 62 °C for 1 min and 95 °C for 10s. A serial dilution of a random sample (4 dilutions, 1:2) was added to each run to create a standard curve to calculate PCR efficiency, and a human genomic DNA reference sample was included to control for inter- and intra-plate variability. All samples, standard dilution, reference sample and non-template control were run in duplicates for each primer set, and each primer set was run on a separate plate. To control for instrumental constrains, runs for TEL and HBG of the same sample were performed the same day. A cut-off for qPCR runs was set with PCR efficiency value reaching 100 ± 15%. Any plate producing an efficiency value out of this range was repeated. Specificity of our qPCR runs were analyzed using the melt curve on each plate. Relative TL were calculated using the ΔΔC_T_ method after correcting for the PCR efficiency according to Pfaffl et al (Pfaffl, 2001). Telomere length in kb was calculated using a human genomic DNA reference of 515 kb (cat # 8918d; Science Cell) that was included on each TL and single copy gene run and calculated as follows: relative (2^-ΔΔC_T_ value)×515. The TL of a diploid cell was then divided by the number of chromosome ends (92) to receive the average TL at each chromosome end ((2^− ΔΔC_T_×515)/92). Inclusion of the reference sample on all PCR runs resulted in non-significant variation and our method showed very high intra- and inter-plate repeatability (ICC >0.98), which was in line with our previous studies (Asghar et al., 2015; Karell et al., 2017; Asghar et al., 2018). Analysis was performed using the ThermoFisher Cloud software.

### Statistical analysis

The study investigated the TL decline at both a population level and an individual level with linear mixed effects models (lme) with a random intercept, and a random slope.

To assess the association between malaria and TL and the difference in change of TL between the two cohorts, we used a linear mixed effect model, adjusted for the potential confounders, age, sex, survey year, genotypes for α-thalassaemia and sickle genotype. The cumulative number of malaria episodes was retrieved from the longitudinal active weekly case detection of malaria in Junju and consists of the number of episodes experienced between the first individual data entrance (2005) and the date of last TL-survey in 2013. We also performed a sub-group analysis for each cohort separately, to evaluate differences in TL distribution. Age was centered at the median (7 years).

To test whether TL may be associated with clinical malaria episodes during the follow-up, we used Cox- regression, assessing the association of TL in year 2007 with future symptomatic malaria infections until the last telomere measurement in 2013.

Statistical analyses were performed in the R statistic software, version 4.1.2 (R Core Team (2020). R Foundation for Statistical Computing, Vienna, Austria. URL: https://www.R-project.org/) (Team TRC, 2018). Graphical presentations were obtained in JMP, version 14.0.

## Results

### Demographics of the study population and prevalence of *P. falciparum* infections

We measured TL in 886 blood samples from three annual cross-sectional surveys for 308 children (Junju: n=218, Ngerenya n=90) and associated them with *P. falciparum* malaria records from a longitudinal surveillance study covering over an average of 11 years for Junju. Demographic data of the children included from both cohorts, and TL measurements for the three cross-sectional surveys, 2007, 2010 and 2013, are presented in **Table 1**; **Supplementary Table 1**, respectively. At the time of the cross-sectional sample collections, most children were healthy; and, collectively, there were a total of seven non-malarial fevers, eight symptomatic malaria infections and 208 asymptomatic *P. falciparum* infections in Junju (19-47% PCR positivity), with the majority of cross-sectional asymptomatic infections (47% PCR positivity) being reported in 2010 (**Supplementary Table 1**). In Ngerenya, a total of 11 non-malaria fevers were reported during the surveys, and none of them had *P. falciparum* parasites detected by qPCR.

**Table 1:** Characteristics of study population.

Variable	Cohort	
	Junju (Moderate transmission)	Ngerenya (No transmission)
Number of children	218	90
Age in years at first TL-survey 2007, median (range)	5 (0-9)	3 (0-7)
Female, n (%)	114 (52.3)	46 (51.1)
Number of TL-surveys per child (2007, 2010, 2013), mean (range)	2.85 (1-3)	2.91 (2-3)
3 TL-surveys, n (%)	187 (85.8)	82 (91.1)
2 TL-surveys, n (%)	31 (14.2)	8 (8.9)
Total number of survey samples for telomere measurement	624	262
Sickle cell typing, n (%) HbAA	175 (80.7)	79 (87.8)
HbAS	42 (19.3)	11 (12.2)
Thalassemia typing, n (%) Normal	77 (35.3)	32 (35.6)
Heterogenous	111 (50.9)	34 (37.8)
Homogenous	30 (13.8)	24 (26.7)
Malaria incidence rate (episodes/child/year)*	1.15	0
Cumulative number of symptomatic malaria episodes until TL survey 2013 (weekly data), mean (range)	6 (1-25)	0
Cumulative number of asymptomatic infections until TL survey 2013 (yearly surveys), mean (range)	2 (0-6)	0

Using active weekly case detection for malaria, a total of 3278 P*. falciparum* malaria cases were detected in Junju children from 2005 to 2013. Malaria was invariably caused by *P. falciparum*, and the parasite densities, evaluated by qPCR, ranged from 1- 162–868 p/µL blood (median= 45). The mean number of malaria fevers between 2005 and the TL survey in 2013 was 7 (range 1-57) per child in Junju. The mean of non-malarial fevers was 5.3 (range 0-16) per child during the surveillance period in Junju, compared to 5.5 (range 0-17) cases of non-malarial fevers in Ngerenya. No cases of *P. falciparum* infection were recorded in Ngerenya.

### Telomere length and host factors

**Figure 2:**
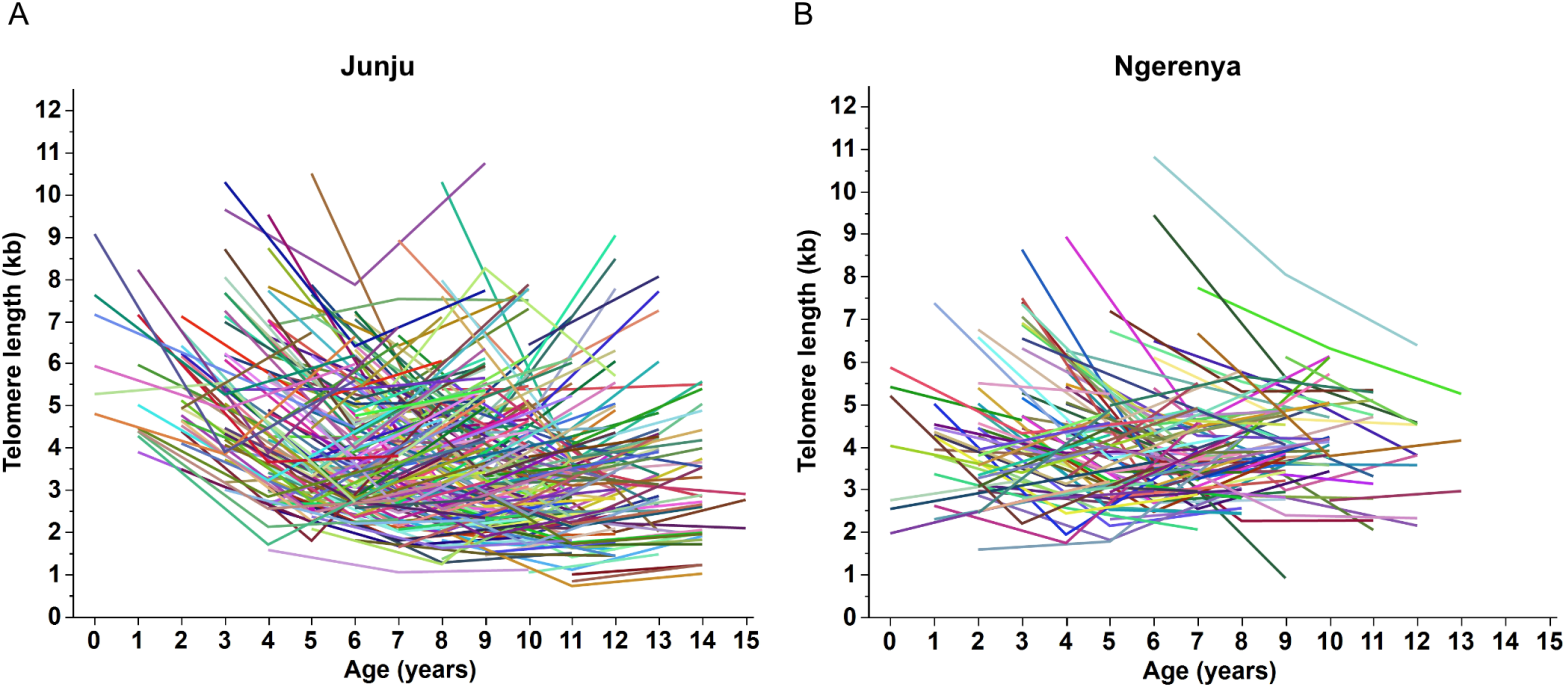
Telomere kinetics in exposed Junju children **(A)** and unexposed Ngerenya children **(B)**.

We measured TL from 624 and 262 samples from Junju and Ngerenya, respectively (**Table 1**; **Figure 2**). Based on the conditional modes of the random slopes, the fitted mixed−effects model suggested an age−related decrease in TL for the majority of children (approximately 85%), with only three individuals exhibiting an estimated positive slope (**Table 2**). The effects of age on TL were less pronounced in individuals with initially shorter telomere kinetics and is interpreted as lower TL shortening rate with time (**Supplementary Figure 1**).

**Table 2:** Effect of host factors on TL.

Telomere length	Est.	SE	df	t	p
Intercept	3.99	0.30	329	13.37	**<0.001**
Age centered at 7	-0.11	0.04	317	-2.93	**0.004**
Sex	0.29	0.15	303	1.84	0.07
Cohort	0.13	0.20	330	0.65	0.52
Survey year 2010	-1.38	0.14	580	-9.84	**<0.001**
Survey year 2013	-0.37	0.25	418	-1.5	0.13
**Cumulative malaria episodes**	0.02	0.01	432	1.23	0.22
Status at survey					
Non-malarial fever	-0.11	0.25	605	-0.43	0.66
Symptomatic malaria	-0.17	0.37	471	-0.48	0.63
Asymptomatic infection	0.22	0.10	703	2.14	**0.03**
Sickle cell trait					
AS	-0.05	0.21	302	-0.22	0.83
SS	-1-64	1.46	248	-1.13	0.26
**Thalassemia**					
Heterozygous	0.25	0.17	303	1.44	0.15
Homozygous	0.45	0.23	299	1.96	0.05

Random effects *σ_Intercept_* = 1.61; *σ_age_* = 0.10; *ρ* = -0.66.

Linear mixed effect model presenting the effect of host and environmental factors on the population TL. The intercept in the model represents TL when all other variables are zero. Survey year 2007 is used as reference in this model. Number of individuals n=308, number of observations n= 883. σ denotes the variance associated with random effects and residual error, while ρ represents the correlation between repeated telomere length measurements within individuals. P values < 0.05 are highlighted in bold text.

On a population level, results from our linear mixed effect model of the effect of host and environmental factors on the population TL show that TL decreases with age (lme, Est.= -0.11, SE = 0.04, t= -2.93, p=0.004; **Table 2**), and was not affected by sex (lme, Est. = 0.29, SE = 0.15, t= 1.84, p =0.067; **Table 2**) or the cohort (lme, Est. = 0.13; SE = 0.20; t= 0.65, p= 0.52; **Table 2**). The interaction between age and sex was non-significant in a separate LME analysis (lme, Est.= -0.01; SE = 0.02; t= -0.07, p=0.94) and thus, was excluded from the main model. Out of the cross-sectional surveys in 2007, 2010 and 2013, population TL was shortest during the survey in 2010 (lme, Est.= -1.38, SE = 0.14; t= -9.84, p<0.001; **Table 2**).

### Telomere length, non-malarial fever and *Plasmodium falciparum* infection at survey

Non-malarial fever at the time of cross-sectional survey was not associated with TL in the cohorts (lme, Est.=-0.11, SE = 0.25, t= -0.43, p=0.66; **Table 2**). Neither did an ongoing febrile malaria episode at time of cross-sectional survey affect TL (lme, Est.=-0.17, SE = 0.37, t= -0.48, p=0.63). However, the asymptomatic *Plasmodium* infections detected at the time of cross-sectional surveys were associated with a modest increase in TL (lme, Est. = 0.22, SE = 0.10, t=2.14, p=0.03; **Table 2**; **Supplementary Figure 2**). Although statistically significant, the effect size was small and the p−value marginal; therefore, the finding should be interpreted cautiously, and its clinical relevance is uncertain.

### Cumulative number of malaria episodes and telomere length

To assess whether previous malaria infections affect TL, we used active surveillance data from children exposed to moderate malaria transmission in Junju to calculate the number of cumulative malaria episodes (fever associated with a *P. falciparum* infection density) per child. During the follow-up period from 2005 to the TL-survey in 2013, an average of six cumulative episodes per child were reported (**Table 1**). The cumulative number of malaria episodes (fever and parasitaemia >0) did not affect TL (lme, Est. = 0.02, SE = 0.01, t=1.23, p=0.22; **Table 2**), adjusting for age. We further ran the same model with a stricter definition of malaria episode, including only fever cases presented with parasitaemia > 2500 p/µl blood, and the outcome was the same (lme, Est. = 0.02, SE = 0.02, t=1.23, p=0.22).

### Telomere length and risk of malaria during follow-up

To evaluate whether TL predicts future symptomatic malaria infection in Junju children, we performed a Cox regression analysis of the TL measured in 2013 and time to the first subsequent malaria episode. As telomere length was measured in bulk peripheral blood leukocytes, the estimates do not reflect telomere attrition or maintenance within specific cell types, but rather the overall state of immune activation in circulating leukocytes at the time of sampling. We therefore adjusted all the regression models for age to account for baseline differences in immune maturation.

Our results show that the 2013-TLs did not predict future malaria episodes when adjusting for age (Cox regression, AdjHR=1.05, p=0.21; **Table 3**). The results remained non-significant when the model was applied to TL measurements in 2007 and 2010 and the subsequent malaria episode data (**Supplementary Table 3**).

**Table 3:** Telomere length and risk of symptomatic malaria during follow-up Junju. Cox regression. Time to first episode after TL-survey 2013.

TL 2013	HR (95%CI)	p	HR age adjusted (95%CI)	p
**All**	1.08 (0.99 – 1.16)	0.06	1.05 (0.96 – 1.14)	0.21
Stratified by Hb				
HbAA	1.11 (1.01 – 1.20)	**0.02**	1.07 (0.98 – 1.18)	0.12
HbAS	0.90 (0.71 – 1.13)	0.38	0.88 (0.69 – 1.29	0.32
Stratified by α-thalassaemia				
Normal	1.09 (0.96 – 1.23)	0.19	1.06 (0.93 – 1.22)	0.38
Heterogeneous	1.20 (1.03 – 1.40)	**0.01**	1.18 (1.01 – 1.37)	**0.03**
Homogenous	0.92 (0.75 – 1.11)	0.38	0.87 (0.71 – 1.07)	0.14
Stratified by parasite positivity at survey 2013				
PCR negative	1.05 (0.96 – 1.16)	0.30	1.03 (0.93 – 1.14)	0.61
PCR positive	1.08 (0.92 – 1.26)		0.33	

P values < 0.05 are highlighted in bold text.

### Telomere length and hemoglobinopathies

Hemoglobinopathies, including sickle cell haemoglobin and α-thalassaemia, have been associated with providing an evolutionary advantage and protection against malaria (Roberts and Williams, 2003). However, there was no difference in TL between children with normal haemoglobin type and carriers for the sickle trait (genotype AS and SS: lme, p >0.05; **Table 2**). Children with homozygous α-thalassaemia appeared to have longer telomeres than children with normal haemoglobin genotype, but with borderline significance in the main model (lme, Est. = 0.45, SE = 0.23, t=1.95, p=0.05; **Table 2**).

### Sub-group analysis within the exposed and unexposed cohorts

**Figure 3:**
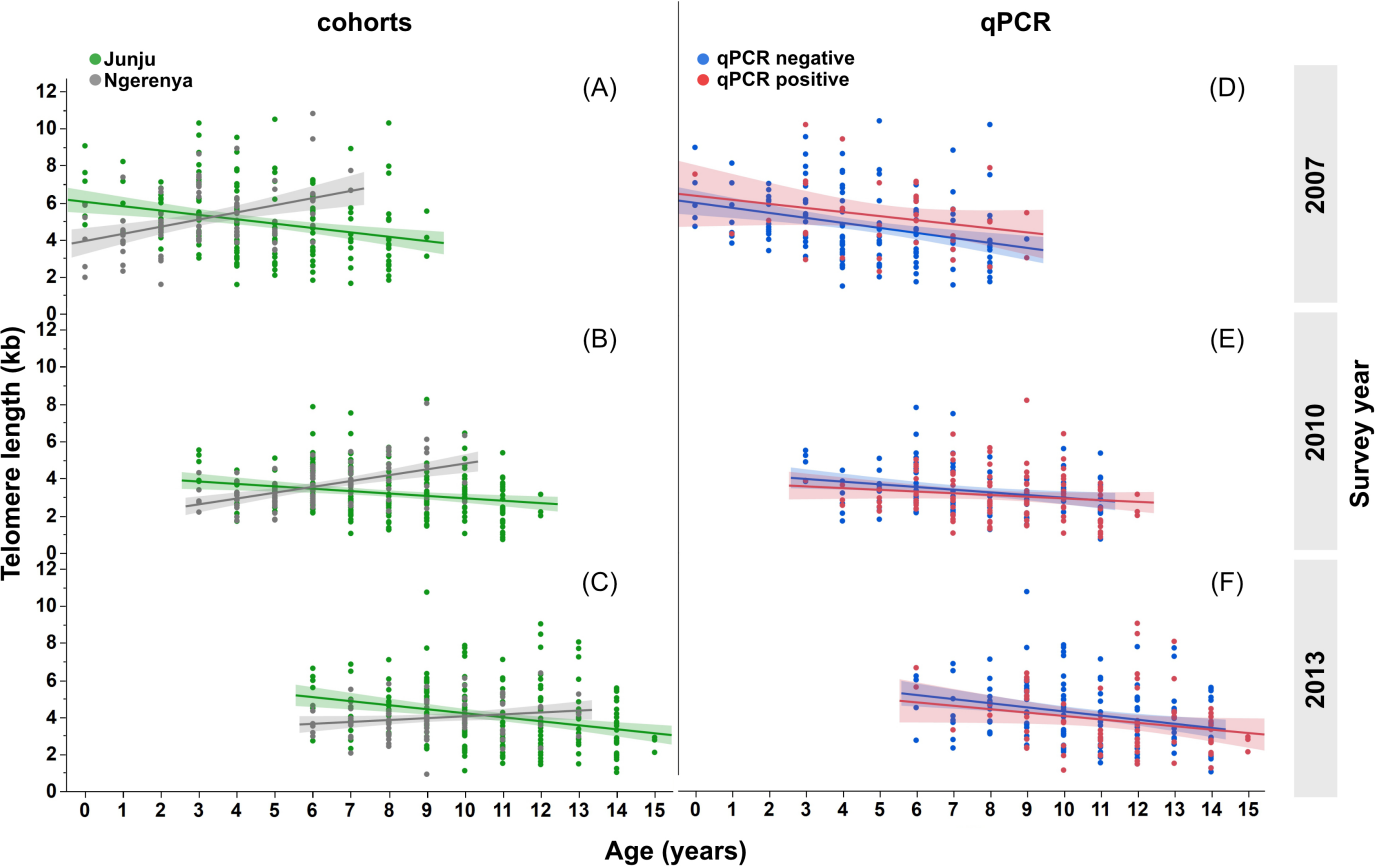
Telomere length with age in exposed vs unexposed children. Graphs are based on repeated cross-sectional TL measurements (at least three per child). **(A-C)** Telomere length distribution with age over time (2007/2010/2013) in children from Junju (green; n=218) and Ngerenya (grey; n=90). **(D-F)** Correlation of TL and age in *Plasmodium* positive Junju children (symptomatic and asymptomatic infection) vs non- infected children in Ngerenya at TL-survey.

We further performed a subgroup analysis (lme) on each cohort separately, which showed that population TL in Ngerenya was differently distributed over age compared to Junju, with more children in Ngerenya presenting a smaller age effect on TL (**Supplementary Table 2**). The subgroup analysis for Junju yielded the same results as in the main model (**Table 2**). However, our results from the subgroup analysis in Ngerenya showed that sex was associated with TL (**Supplementary Table 2**), and that Ngerenya children who carry the genotype for α-thalassaemia had longer telomeres compared to children with normal haemoglobin genotype (**Supplementary Table 2**). Furthermore, we found that TL in Ngerenya was reduced in the survey-years, 2010 and 2013 (**Figure 3**). Furthermore, TL was shorter in both cohorts in 2010, though with a more pronounced effect in Ngerenya (**Figure 3**). Collectively, these findings suggest there were other, non-malaria-related factors affecting TL.

## Discussion

Here, we assessed the effect of cumulative malaria episodes and *P. falciparum* infections on telomere length (TL) in children (aged <1–15 years) from two culturally similar cohorts of children in Kenya: one exposed to moderate malaria transmission and the other with very little *P. falciparum* transmission (Ndungu et al., 2012). A key strength of this study is the long−term, detailed longitudinal malaria surveillance; however, TL was assessed at three time points, each three years apart, limiting the ability to detect short−term telomere shortening and recovery during acute inflammatory episodes, as reported in malaria−naïve adults in our controlled human malaria infection (CHMI) study (Miglar et al., 2021). Within these constraints, we found no evidence that cumulative symptomatic malaria episodes were associated with shorter TL over time, nor did TL differ between exposed and unexposed children. Asymptomatic *P. falciparum* parasitemia at the time of sampling was associated with slightly longer TL, although this effect was modest and supported only marginally by the data (Coef. Est. 0.22).

These findings should be interpreted in the context of prior studies rather than as direct contradictions. McQuillan et al. reported a negative association between TL and malaria endemicity in adults across sub-Saharan Africa based on cross−sectional data spanning a wide age (McQuillan et al., 2024), whereas our study focused on children under active surveillance for febrile malaria, enabling age−matched longitudinal comparisons during early life, when hematopoietic turnover is high and TL dynamics are most pronounced (Aviv et al., 2009). Moreover, while McQuillan et al. assessed population−level endemicity across culturally and behaviorally diverse adult populations, our cohorts from Junju and Ngerenya share similar socioeconomic and environmental conditions, reducing lifestyle−related confounding (Ndungu et al., 2012; Illingworth et al., 2013). Together, these differences in age, study design, and analytical approach likely explain the distinct patterns observed.

Similarly, our previous studies in Swedish travellers experiencing a single naturally acquired malaria episode, and in malaria−naïve adults undergoing CHMI, demonstrated transient TL shortening following infection (Asghar et al., 2018; Miglar et al., 2021). In the present study, the limited number of symptomatic infections at sampling and the long interval between TL measurements limited the assessment of such short−term dynamics. Taken together, these findings suggest that *P. falciparum* infection can transiently influence TL during acute inflammatory responses, while longer−term TL trajectories in children living in endemic settings are primarily driven by age and baseline TL rather than cumulative malaria exposure. The mechanisms underlying transient TL shortening and subsequent restoration remain incompletely understood. While inflammation−driven oxidative stress has been implicated in accelerated TL attrition in malaria−naïve adults (Miglar et al., 2021), telomerase activity was not assessed in the present study, and previous work has shown no direct correlation between TL restoration and telomerase expression in this context (Asghar et al., 2018; Miglar et al., 2021). Consistent with this, emerging evidence indicates that telomerase activity and TL are not interchangeable biomarkers *in vivo*, as discordant patterns have been observed across multiple biological systems (Asghar et al., 2018; Miglar et al., 2021). Together, these observations support the idea that TL regulation may involve mechanisms beyond telomerase. Telomerase−independent pathways, including recombination−based alternative lengthening of telomeres and telomere−associated RNA processes (e.g. TERRA), have been described and remain important candidates for future work in non−malignant settings (O’Sullivan and Greenberg, 2025).

One possible explanation for the apparent discrepancy with our earlier studies is the limited temporal resolution in the current study, where TL was measured only at three-year intervals. Alternatively, differences in antimalarial immunity may account for the divergence. Participants in the CHMI and travelers’ studies were malaria-naïve, and their infections triggered pronounced inflammation and oxidative stress, recently linked to accelerated telomere shortening (Miglar et al., 2021). In contrast, the children in our study live in an endemic area and therefore likely possess substantial anti-parasite and immunoregulatory responses (Illingworth et al., 2013; Tran et al., 2019), resulting in lower levels of cellular activation and inflammation at the time of sampling (Langhorne et al., 2008).

Compared with 2007 and 2013, we observed shorter TLs in 2010. This trend appears to correlate with higher rates of febrile malaria in Junju that year. However, it does not explain the similar reductions in TL observed in Ngerenya, where *P. falciparum* transmission had declined to very low levels by 2010 (Ndungu et al., 2012; Illingworth et al., 2013). This suggests that other environmental factors common to both cohorts may have contributed.

We detected a marginal but positive effect of asymptomatic *P. falciparum* parasitaemia on TL at the time of survey. A possible explanation is the development of malaria-specific tolerance mechanisms. A regulatory immune state is commonly observed in individuals living in endemic regions, including malaria-naïve adults undergoing sequential CHMI, and is thought to minimize immunopathology (Illingworth et al., 2013; Tran et al., 2019; Dooley et al., 2023). A recent study by Frimpong et al. showed that senescence markers in T cells from Ghanaian children with asymptomatic *P. falciparum* infection did not differ from those in healthy controls (Frimpong et al., 2019). Furthermore, asymptomatic infections are associated with transcriptional signatures indicating repression of inflammation and cellular proliferation via pathways involving CTLA-4 and p53 (Tran et al., 2019; Studniberg et al., 2022). This immunosuppressive profile likely reduces cellular turnover and oxidative stress, which may explain why asymptomatically infected children in our study exhibited longer telomeres than uninfected children. Nevertheless, we did not measure leukocyte subset proportions, telomerase activity, or cell−specific telomere lengths in this study; therefore, we cannot distinguish reduced telomere attrition from compositional effects or other immune−regulatory processes. Future studies with cell−type–resolved TL measurements and concurrent immunophenotyping across symptomatic and asymptomatic states will be needed to test these hypotheses.

We expected to detect an effect of sickle haemoglobin (HbS) on TL, as sickle cell trait is a well-established malaria-protective factor (Roberts and Williams, 2003; Wambua et al., 2006). Under the hypothesis that malaria accelerates TL attrition, we anticipated that children with normal haemoglobin genotype would have shorter TLs than those carrying HbAS. However, despite intensive longitudinal surveillance capturing nearly all malaria-related morbidity, we found no influence of HbS trait on TL. Similarly, we were unable to explain the trend toward longer TLs in children with homozygous α-thalassemia in Ngerenya. α-thalassemia (both homozygous and heterozygous) is associated with reduced malaria-related hospital admissions and lower rates of severe disease and anemia in Kenyan children (Wambua et al., 2006). Nevertheless, the TL effect was more marked in Ngerenya, the malaria-free site, and was observed in only a few individuals, making the finding uncertain.

A limitation of this study is the sparse TL measurement schedule - three time points, each three years apart - which may have prevented detection of finer TL fluctuations. Additionally, sample sizes for several comparisons were small, limiting statistical power. The study was also confined to a single endemic region, yet parasite–host interactions vary substantially with transmission intensity. Nonetheless, a major strength is the availability of detailed longitudinal surveillance data (averaging 11 years per participant), including cumulative malaria episodes across two transmission settings.

Our findings differ from those of studies in birds chronically infected with malaria, which showed accelerated TL shortening and reduced lifespan (Asghar et al., 2015). In birds, both absolute TL and the rate of shortening predict life expectancy (Haussmann et al., 2003; Asghar et al., 2015; Spurgin et al., 2018; Whittemore et al., 2019). However, avian malaria pathophysiology differs from that in humans: birds harbour lifelong chronic parasitemia, which may drive sustained cellular activation, and they possess nucleated erythrocytes and are infected by different *Plasmodium* species.

In conclusion, this is the first study to examine the effects of repeated malaria episodes and chronic asymptomatic *P. falciparum* infection on TL dynamics over time in individuals living in an endemic human population. We show that although TL shortens with age, these changes are likely independent of persistent malaria episodes and *P. falciparum* exposure. While we applied a main−effects approach in this study, interactions between predictors - such as age, baseline TL, and malaria exposure - may contribute to TL dynamics and remain to be explored in future studies with greater temporal resolution. Furthermore, larger studies across diverse transmission settings, with more frequent TL measurements and longer follow-up, including adults, are needed to confirm our findings.

## Data Availability

The raw data supporting the conclusions of this article will be made available by the authors, without undue reservation.
