# Supplementary material for: Cumulative Plasmodium falciparum infections do not drive long-term telomere shortening in Kenyan children
Source: Front Cell Infect Microbiol. 2026 May 13;16:1750881. doi: 10.3389/fcimb.2026.1750881 (PMC13212443; doi:10.3389/fcimb.2026.1750881)
Supplement: Supplementary file 1 [file Table1.docx]

**Supplementary tables**

**Table S1. Association of malaria and telomeres in study population**

|  | **Junju** | | | **Ngerenya** | | |
| --- | --- | --- | --- | --- | --- | --- |
|  | *2007* | *2010* | *2013* | *2007* | *2010* | *2013* |
| Number of children | 190 | 218 | 219 | 83 | 89 | 90 |
| Age in years, mean (range) | 5 (0-9) | 8 (3-12) | 11 (6-15) | 3 (0-7) | 6 (3-10) | 9 (6-13) |
| Telomere length in kb, mean (range) | 4.9 (1.6-10.5) | 3.2 (0.7-8.3) | 4 (1-10.7) | 5.1 (1.6-10.8) | 3.6 (1.7-8) | 4 (0.9-6.4) |
| Asymptomatic *Plasmodium* infections at survey, n (%)˚ | 36 (19) | 102 (47) | 70 (32) | 0 | 0 | 0 |
| Fever at survey, n (%) | 6 (3.2) | 8 (3.7) | 1 (0.5) | 4 (4) | 5 (4.8) | 2 (2) |
| Malaria | 2 (33.3) | 6 (75) | 0 | 0 | 0 | 0 |
| Non-malarial fever | 4 (66.7) | 2 (25) | 1 (100) | 4 | 5 | 2 |
| Cumulative number of symptomatic infections before survey,  mean (range)* | 0.78 (0-5) | 5.2 (0-17) | 9.2 (0-27) | 0 | 0 | 0 |
| Cumulative number of non-malaria fevers before survey, mean (range)* | 1.2 (0-8) | 3.4 (0-16) | 4.9 (0-21) | 2 (0-12) | 4.3 (0-14) | 5.3 (0-16) |
| Cumulative number of symptomatic infections between surveys  (2007-2013), mean (range)* | 10.2 (0-33) |  |  | 0 | 0 | 0 |
| Cumulative number of symptomatic infections after surveys, mean (range)* | 15.24 (1-60) | 10.35 (0-50) | 3.8 (0-16) | 0 | 0 | 0 |
| ˚based on qPCR |  |  |  |  |  |  |
| *based on weekly surveillance data from entry to the cohort study to exit at age 15 |  |  |  |  |  |  |

**Table S2. Factors affecting telomere length in 2013.**

|  | **Junju** | | | | | | | | | |  |
| --- | --- | --- | --- | --- | --- | --- | --- | --- | --- | --- | --- |
|  | unadjusted | | | | | | adjusted for age | | | | |
|  | | Coef. | SE | t | p | 95% CI | Coef. | SE | t | p | 95% CI |
| **Age at baseline** | | -0.22 | 0.05 | -4.25 | **<0.001** | -0.32 – -0.12 |  |  |  |  |  |
| **Sex** | | -0.09 | 0.23 | -0.40 | 0.69 | -0.55 – 0.37 | -0.11 | 0.23 | -0.51 | 0.61 | -0.56 – 0.33 |
| HbAA vs HbAS | | -0.21 | 0.30 | -0.69 | 0.49 | 0.80 – 0.38 | -0.12 | 0.29 | -0.41 | 0.69 | -0.69 – 0.45 |
| **Thalasseamia** | |  |  |  |  |  |  |  |  |  |  |
| Heterogenous | | 0.16 | 0.26 | 0.62 | 0.54 | -0.35 – 0.66 | 0.07 | 0.25 | 0.28 | 0.78 | -0.42 – 0.56 |
| Homogenous | | 0.38 | 0.37 | 1.02 | 0.31 | -0.35 – 1.11 | 0.46 | 0.36 | 1.28 | 0.20 | -0.25 – 1.17 |
| **PCR parasite positive at survey 2013** | | -0.36 | 0.25 | -1.45 | 0.15 | -0.85 – 0.13 | 0.20 | 0.24 | -0.83 | 0.41 | -0.68 – 0.28 |
| **Cumulative number of symptomatic malaria** | |  |  |  |  |  |  |  |  |  |  |
| Fever and parasites >0 at weekly visit | | 0.01 | 0.02 | 0.33 | 0.74 | -0.04 – 0.05 | -0.02 | 0.02 | -0.86 | 0.39 | -0.07 – 0.03 |
| Fever and parasites >2500 at weekly visit | | 0.02 | 0.03 | 0.78 | 0.44 | -0.03 – 0.07 | -0.02 | 0.03 | -0.59 | 0.55 | -0.07 – 0.04 |
| Parasites >0 at weekly visit | | 0.01 | 0.02 | 0.30 | 0.77 | -0.04 – 0.05 | -0.02 | 0.02 | -0.93 | 0.35 | -0.06 – 0.02 |
| Parasites >2500 at weekly visit | | 0.02 | 0.03 | 0.79 | 0.43 | -0.03 – 0.07 | -0.02 | 0.03 | -0.58 | 0.56 | -0.07– 0.04 |
| **Cumulative number of non-malaria fever**˚ | 0.01 | | 0.03 | 0.40 | 0.69 | -0.05 – 0.07 | -0.03 | 0.03 | -1.13 | 0.26 | -0.09 – 0.25 |
| **Cumulative number of asymptomatic infections at yearly surveys^*^** | -0.22 | | 0.06 | -3.32 | **0.001** | -0.34 – -0.09 | -0.13 | 0.07 | -1.89 | 0.06 | -0.26 – 0.01 |
| **Time to previous malaria episode before TL 2013** | -0.001 | | 0.001 | -1.85 | 0.07 | -0.001 - 0.0001 | -0.001 | 0.001 | -1.18 | 0.24 | -0.0006 - 0.0002 |
|  |  | |  |  |  |  |  |  |  |  |  |
| *based on yearly surveillance data from entry to the cohort study to TL survey 2013 | | | | | | | | | | | |
| ˚based on yearly surveys from entry to the cohort study to TL survey 2013 | | | | | | | | | | | |
|  | **Ngerenya** | | | | | | | | | | |
|  | unadjusted | | | | | | adjusted fro age | | | | |
|  | Coef. | | SE | t | p | 95% CI | Coef. | SE | t | p | 95% CI |
| **Age at baseline** | 0.10 | | 0.06 | 1.76 | 0.08 | -0.01 – 0.22 |  |  |  |  |  |
| **Sex** | -0.55 | | 0.20 | -2.71 | **0.01** | -0.95 – -0.15 | -0.51 | 0.20 | -2.25 | **0.01** | -0.92 – -0.11 |
| **HbAA vs HbAS** | 0.18 | | 0.32 | 0.57 | 0.57 | -0.46 – 0.82 | 0.12 | 0.32 | 0.36 | 0.72 | -0.52 – 0.75 |
| **Thalasseamia** |  | |  |  |  |  |  |  |  |  |  |
| Heterogenous | 0.14 | | 0.25 | 0.57 | 0.57 | -0.35 – 0.63 | 0.12 | 0.24 | 0.48 | 0.63 | -0.37 – 0.60 |
| Homogenous | 0.35 | | 0.27 | 1.32 | 0.19 | -0.18 – 0.89 | 0.32 | 0.27 | 1.21 | 0.23 | -0.21 – 0.85 |
| **Cumulative number of non-malaria fevers until TL 2013** | 0.14 | | 0.12 | 1.17 | 0.25 | -0.1 - 0.38 | 0.16 | 0.12 | 1.31 | 0.19 | -0.08 – 0.39 |

**Table S3. Telomere length and risk of symptomatic malaria during follow-up in Junju.** Cox regression. Time to first episode after TL survey in 2007 and 2010.

| **TL 2007 – time to infection after TL-survey 2007** | | | | | **TL 2010 – time to infection after TL-survey 2010** | | | |
| --- | --- | --- | --- | --- | --- | --- | --- | --- |
|  | **HR**  **(95% CI)** | **p** | **HR age adjusted**  **(95% CI)** | **p** | **HR  (95% CI)** | **p** | **HR age adjusted**  **(95% CI)** | **p** |
| **All** | 0.96 (0.89 – 1.04) | 0.30 | 0.94 (0.86 – 1.02) | 0.13 | 1.09 (0.98 – 1.21) | 0.10 | 1.03 (0.92 – 1.15) | 0.66 |
| **Stratified by Hb** |  |  |  |  |  |  |  |  |
| HbAA | 0.96 (0.88 – 1.05) | 0.42 | 0.95 (0.87 – 1.05) | 0.31 | 1.08 (0.96 – 1.21) | 0.19 | 1.02 (0.90 – 1.15) | 0.76 |
| HbAS | 0.96 (0.81 – 1.13) | 0.60 | 0.93 (0.78 – 1.11) | 0.42 | 1.14 (0.87 – 1.51) | 0.33 | 1.17 (0.88 – 1.56) | 0.29 |
| **Stratified by Thalassemia** |  |  |  |  |  |  |  |  |
| Normal | 1.59 (0.92 – 1.19) | 0.50 | 1 (0.86 – 1.16) | 1 | 1.04 (0.88 – 1.23) | 0.61 | 0.95 (0.79 – 1.16) | 0.63 |
| Heterogeneous | 0.96 (0.86 – 1.07) | 0.47 | 0.95 (0.84 – 1.06) | 0.35 | 1.07 (0.91 – 1.26) | 0.40 | 1.03 (0.87 – 1.21) | 0.74 |
| Homogenous | 0.83 (0.66 – 1.05) | 0.12 | 0.82 (0.64 – 1.04) | 0.10 | 1.22 (0.95 – 1.55) | 0.11 | 1.18 (0.91 – 1.54) | 0.21 |
| **Stratified by parasite positivity at survey 2007** | |  |  |  |  |  |  |  |
| PCR negative | 0.96 (0.88 – 1.04) | 0.33 | 0.95 (0.87 – 1.04) | 0.27 | 1.02 (0.89 – 1.19) | 0.70 | 0.96 (0.81 – 1.13) | 0.61 |
| PCR positive | 1.04 (0.86 – 1.26 ) | 0.67 |  |  | 1.12 (0.96 – 1.32 ) | 0.14 |  |  |

**Supplementary figures**

**Figure S1.** Telomere length with age in males (blue) and females (red) in (A-C) Junju and (D-F) Ngerenya at TL-surveys.

**
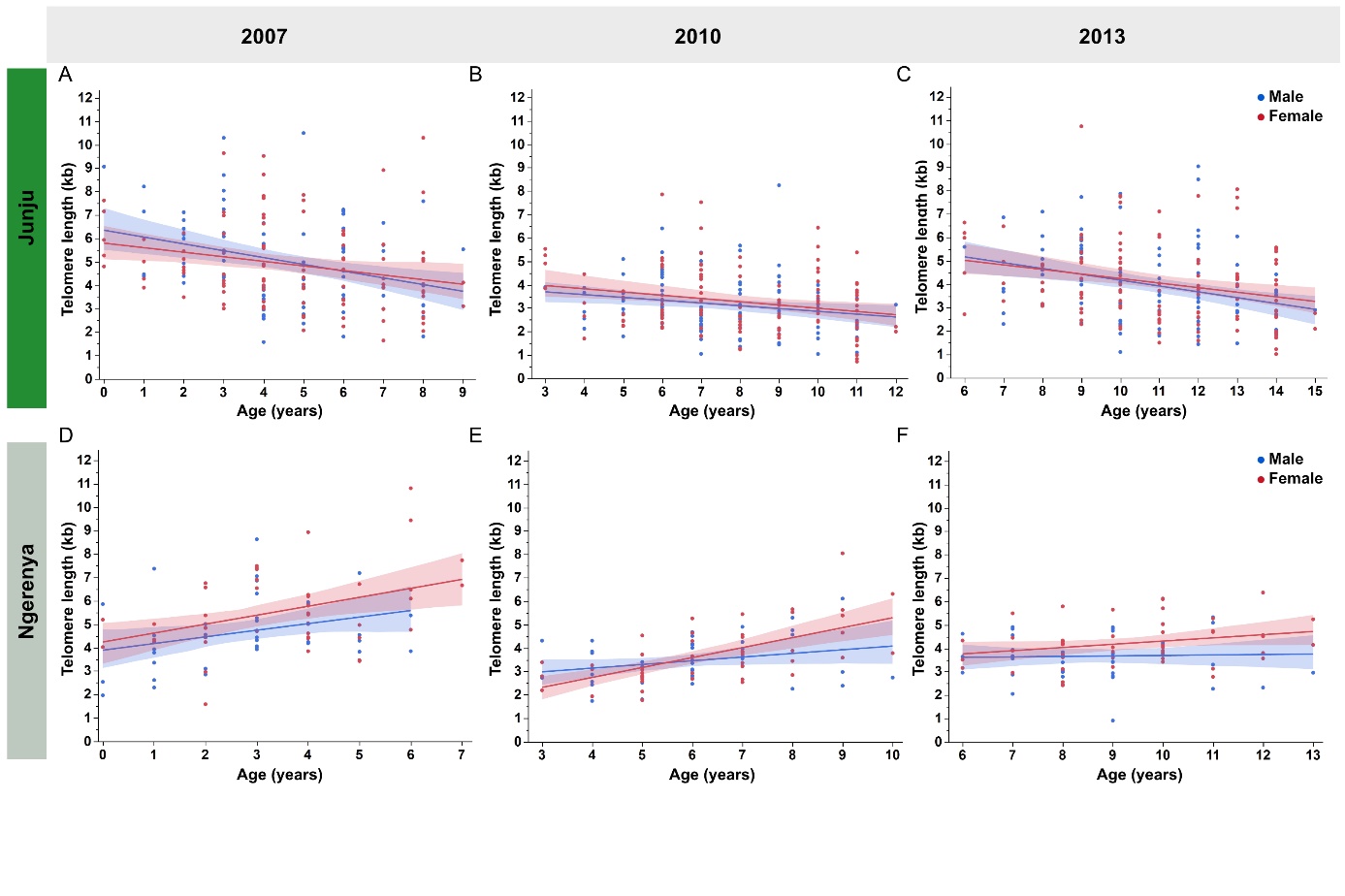
**

**Figure S2.** Relationship of TL and asymptomatic *Plasmodium* infection.

**
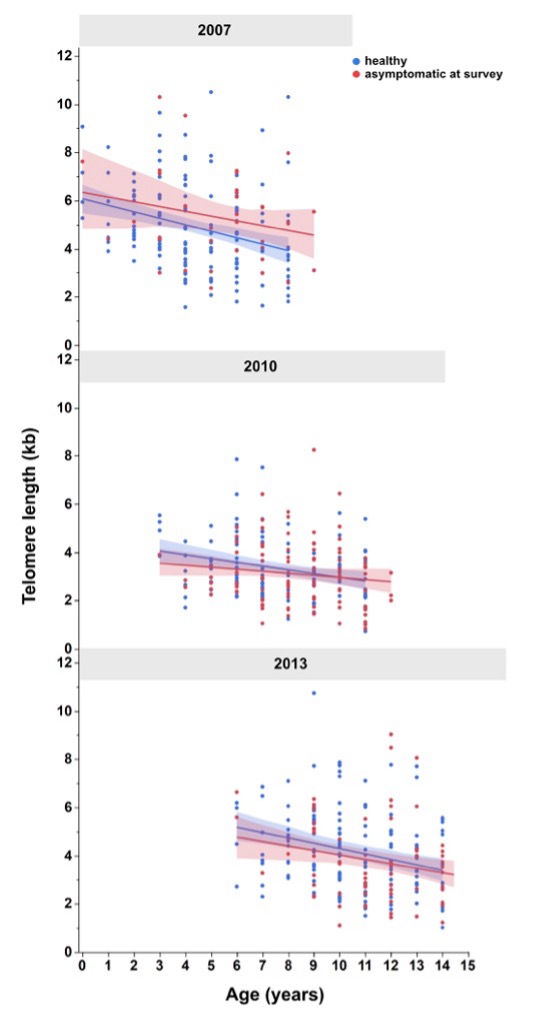
**

**Figure S3.** Correlation of parasite density (log) and TL in infected children at TL-surveys in Junju.

**
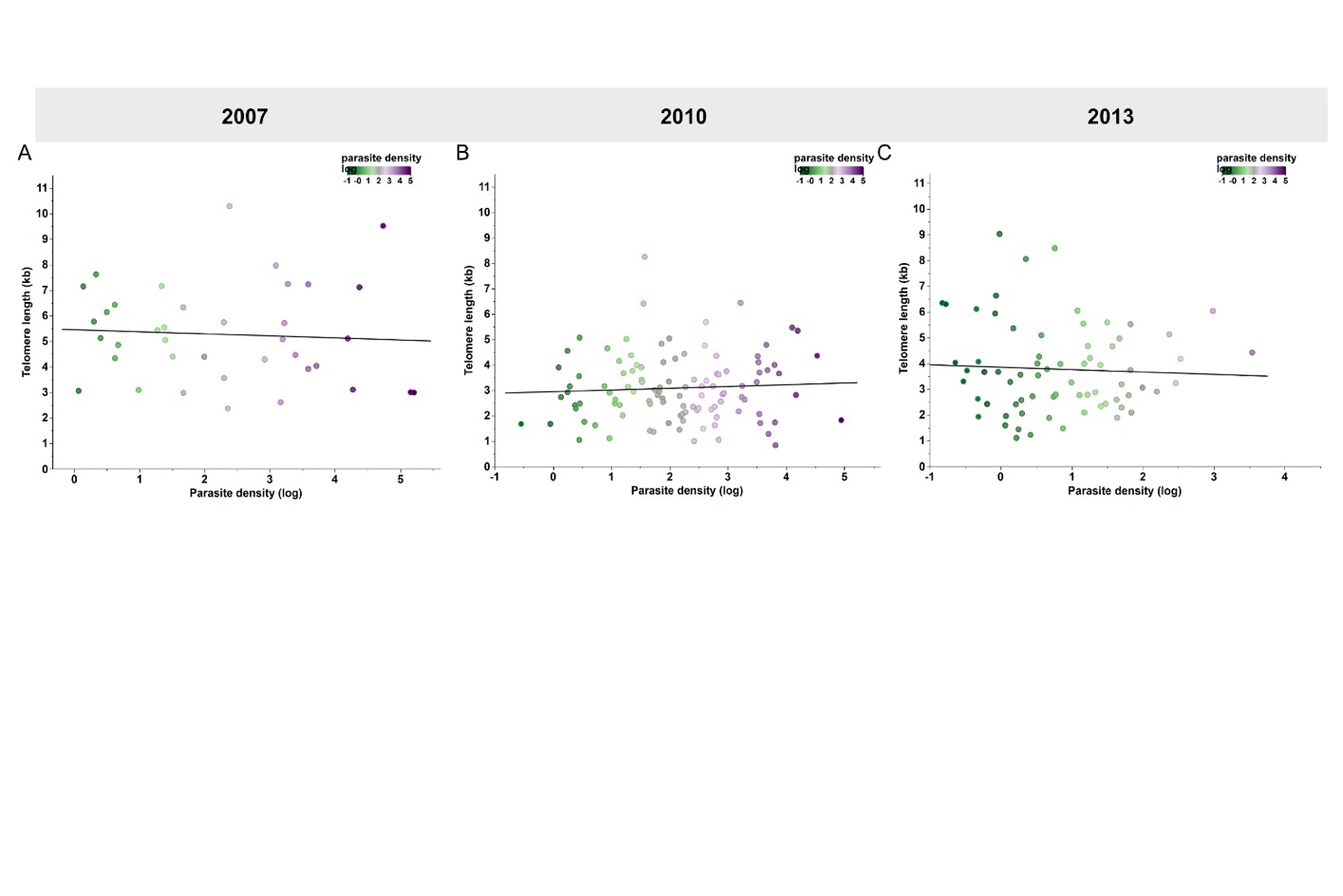
**

**Figure S4.** Telomere length with age across sickle cell genotypes in Junju and Ngerenya for each TL-survey.

**
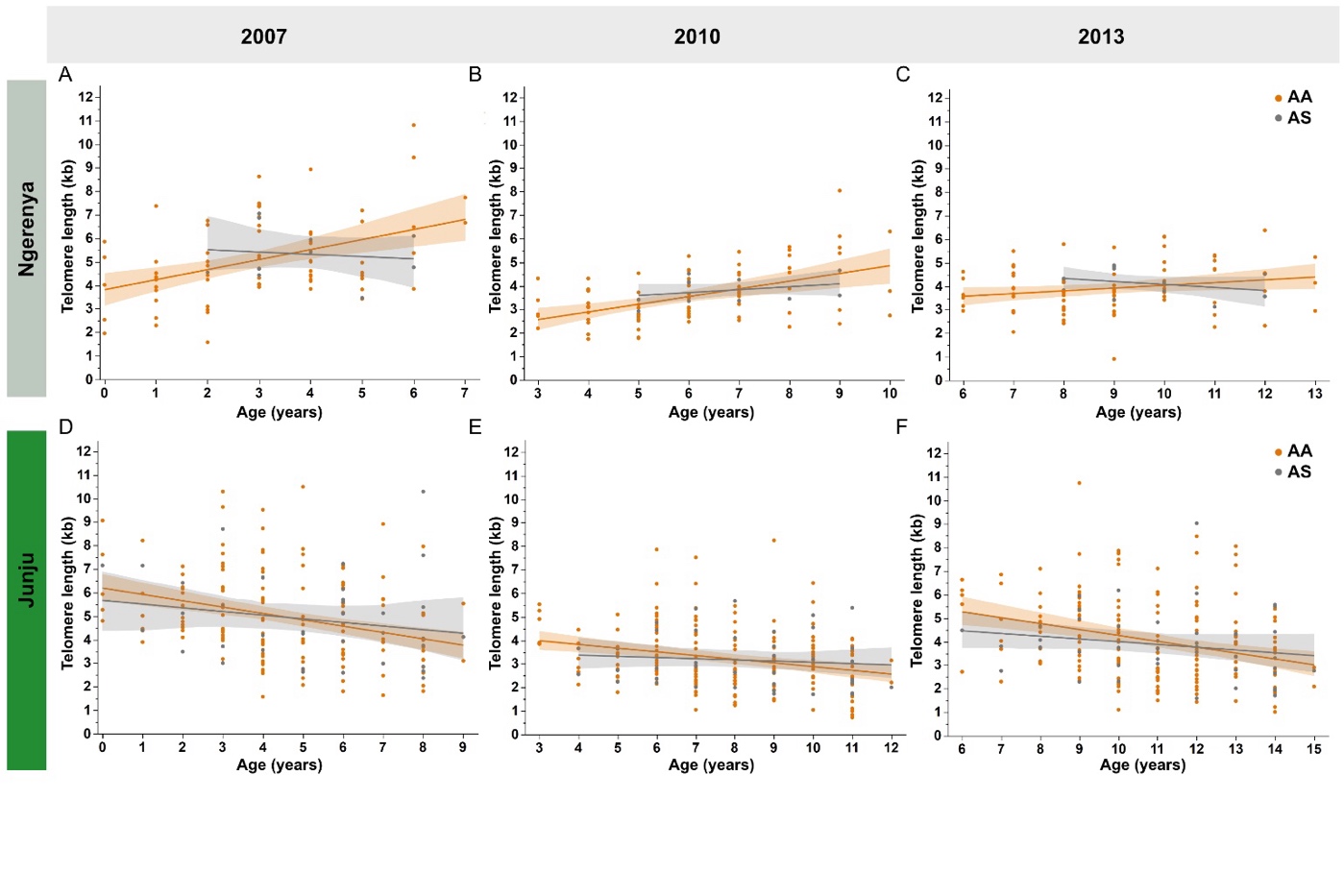
**

**Figure S5.** Telomere length with age across thalasseamia genotypes in Junju and Ngerenya for each TL-survey.

**
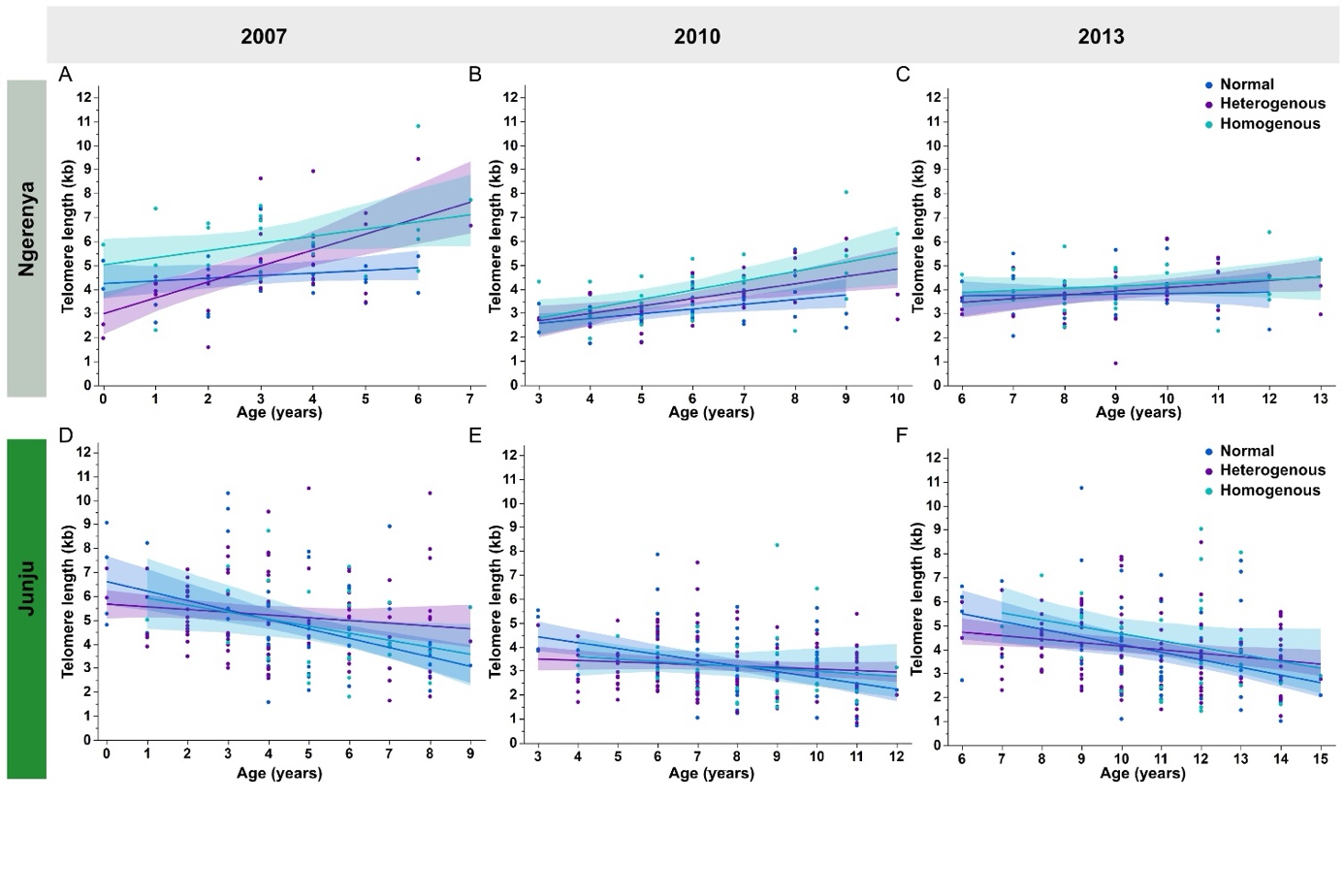
**
